# Survival analysis of clear cell renal cell carcinoma based on radiomics and deep learning features from CT images

**DOI:** 10.1097/MD.0000000000040723

**Published:** 2024-12-20

**Authors:** Zhennan Lu, Sijia Wu, Dan Ni, Meng Zhou, Tao Wang, Xiaobo Zhou, Liyu Huang, Yu Yan

**Affiliations:** a Department of Equipment, Affiliated Hospital of Nanjing University of Chinese Medicine (Jiangsu Province Hospital of Chinese Medicine), Nanjing, Jiangsu, China; b School of Life Science and Technology, Xidian University, Xi’an, Shaanxi, China; c Affiliated Drum Tower Hospital, Medical School of Nanjing University, Nanjing, Jiangsu, China; d The Department of Radiology, Shaanxi Provincial People’s Hospital, Xi’an, Shaanxi, China; e Center for Computational Systems Medicine, School of Biomedical Informatics, The University of Texas Health Science Center at Houston, Houston, TX.

**Keywords:** clear cell renal cell carcinoma, computed tomography, deep learning, radiomics, survival analysis

## Abstract

**Purpose:**

To create a nomogram for accurate prognosis of patients with clear cell renal cell carcinoma (ccRCC) based on computed tomography images.

**Methods:**

Eight hundred twenty-two ccRCC patients with contrast-enhanced computed tomography images involved in this study were collected. A rectangular region of interest surrounding the tumor was used to extract quantitative radiomics and deep-learning features, which were filtered by Cox proportional hazard regression model and least absolute shrinkage and selection operator. Then the selected features formed a fusion signature, which was assessed by Cox proportional hazard regression model method, Kaplan–Meier analysis, receiver operating characteristic curves, and concordance index (C-index) in different clinical subgroups. Finally, a nomogram constructed with this signature and clinicopathologic risk factors was assessed by C-index and survival calibration curves.

**Results:**

The fusion signature performed better than the radiomics signature. Then we combined this signature and 2 clinicopathologic risk factors. This nomogram showed an increase of about 20% in C-index values when compared to clinical nomogram in both datasets. Its prediction probability was also in good agreement with the actual ratio.

**Conclusion:**

The proposed fusion nomogram provided a noninvasive and easy-to-use model for survival prognosis of ccRCC patients in future clinical use, without the requirement to perform a detailed segmentation for radiologists.

## 1. Introduction

Clear cell renal cell carcinoma (ccRCC), constitutes about 70% of primary renal neoplasms.^[1,2]^ Patients diagnosed with ccRCC tend to have a poorer prognosis than chromophobe RCCs and papillary RCCs.^[3,4]^ In addition, many postoperative patients have a recurrence of ccRCC and the prognosis for progression-free survival is poor.^[5]^ Therefore, accurate prediction of survival probability for patients with ccRCC is very helpful for its treatment.

Recently, radiomics features extracted from computed tomography (CT) images provided valuable information to solve various ccRCC-related clinical problems, such as preoperative prediction of nuclear grades,^[6]^ Grading of WHO/ISUP score,^[7]^ determination of PBRM1 mutation status,^[8]^ differentiation of the histopathological grades,^[9]^ and evaluation of Fuhrman grade.^[10]^ However, most of these image studies required radiologists to manually delineate the exact tumor region, which brought heavy workloads for radiologists. Moreover, deep-leaning algorithms could capture high-dimensional characteristics covered in the medical images, which avoid the requirement of detailed tumor segmentation and have proved their usefulness in end-to-end kidney cancer diagnosis,^[11]^ ccRCC nuclear grade prediction,^[12]^ ccRCC identification from oncocytoma,^[13]^ and renal cancer subtyping.^[14]^ Considering the advantages of above 2 methods, we combined them in this study to build a fusion signature for survival prognosis. This is an attempt to achieve a relatively high prediction result and also to reduce the radiologists’ workloads in tumor segmentation simultaneously.

To complete the above objective, multiple steps were designed for a survival nomogram based on the CT images, including the segmentations of region of interest (ROI). The ROI involved in this study is a rough rectangular region surrounding the tumor delineated by experienced radiologists. It reduces the workloads of radiologists, and reaches a good performance by combining traditional low-dimensional radiomics features and complex deep-learning features.

## 2. Materials and methods

### 2.1. Patient samples

**Figure 1.**
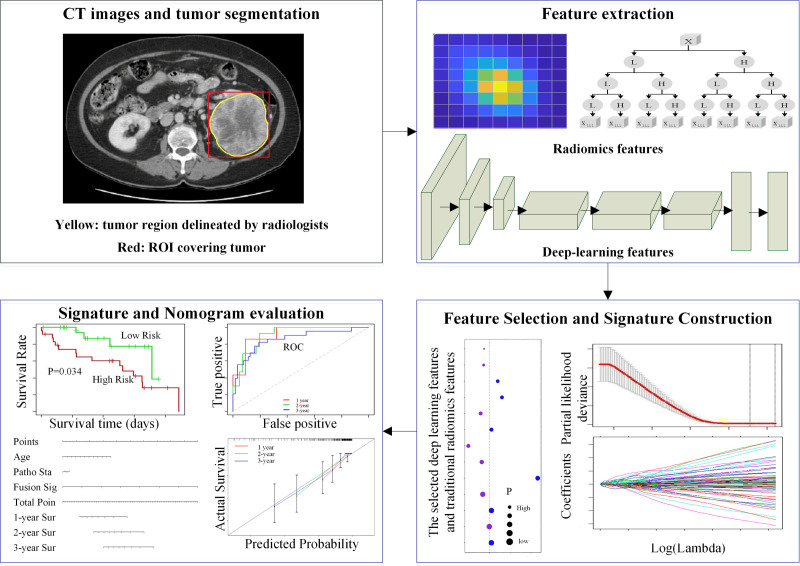
The flowchart of this study. It includes image segmentation, extraction and selection of radiomics and deep-learning features, signature construction, nomogram development, and evaluation of the signatures and nomogram.

In this study, 670 ccRCC patients were enrolled from the Affiliated Hospital of Xuzhou Medical University between October 2012 and March 2021. They were randomly divided into a training dataset and an internal test dataset in the ratio of 3:1. The 152 patients (Clear Cell Renal Cell Carcinoma of the Cancer Genome Atlas) used for external independent validation involved in this study were all obtained from the Cancer Imaging Archive (https://www.cancerimagingarchive.net/). This retrospective study was approved by the institutional ethical committee. Patients involved in this study met the following criteria: (i) histologically confirmed clear cell RCC, (ii) complete records of patients’ survival data, and (iii) available contrast-enhanced CT images scanned in the nephrographic phase. We used images in the nephrographic phase to extract features for survival analysis of ccRCC, since the nephrographic phase is the best phase to detect subtle parenchymal lesions,^[15]^ and there are enough images in this phase. The overall flowchart of this study is shown in Figure 1.

### 2.2. Clinical data analysis

We performed 2 kinds of statistical analyses on these clinical data. First, we applied ANOVA method to evaluate the differences in the clinical variables between training and test cohorts to confirm the randomness of their division. Second, we used Cox regression model to analyze the effects of all possible clinical factors on survival. This analysis takes the survival outcomes and survival time as dependent variables to find the clinicopathologic risk factors that have a significant impact on the survival of ccRCC patients. Two-sided *P*-values of <.05 were considered to indicate significant differences. The processes were implemented in IBM SPSS software (https://www.ibm.com/analytics/spss-statistics-software).

### 2.3. Tumor segmentation

The ROI for this study is a rectangular region surrounding the exact tumor which was then resized to a size of 227 × 227 (pixels × pixels). This rectangular ROI contained the tumor and part of parenchyma region around the tumor, which can be easily drawn by radiologists. On the contrary, a precise tumor region was also used in the following analysis for comparison, which was delineated manually by 2 experienced radiologists separately in the format of DICOM using the 3D-slicer (https://www.slicer.org) open-source software.^[16]^

### 2.4. Traditional radiomics features extraction and selection

After tumor segmentation, 849-dimensional radiomics features were extracted from the labeled pixels inside the rectangular ROI and the exact tumor region by “pyradiomics” package in Python (https://pyradiomics.readthedocs.io/, v3.6.0). The detailed feature extraction process was shown in Supplementary File, Supplemental Digital Content, http://links.lww.com/MD/O223. They can be divided into 4 categories: (1) first-order statistical features describing the gray-scale distribution of pixels,^[17]^ (2) shape features quantifying the shape and size of the tumor area, (3) textural features estimating the uniformity, heterogeneity, coarse degree, etc,^[18]^ and (4) wavelet features calculated on the images preprocessed using wavelet filters (Supplementary File.pdf, Supplemental Digital Content, http://links.lww.com/MD/O223).

To select the most significantly survival-related radiomics features, the 849-dimensional features extracted from the 2 kinds of ROIs were then processed by Cox proportional hazard regression model (Cox) with the criterion of *P* < .05 and the least absolute shrinkage and selection operator (LASSO) sequentially. The detailed feature selection process was shown in Supplementary File, Supplemental Digital Content, http://links.lww.com/MD/O223. The whole analysis process was implemented by in-house R scripts(https://cran.r-project.org).

### 2.5. Deep-learning features extraction and selection

Due to relatively insufficient medical images, we performed transfer learning to analyze these CT images using AlexNet with corresponding pretrained weights.^[19]^ The rectangular region surrounding the whole tumor was first resized to a square area with a size of 227 × 227 (pixels × pixels). Then from the square ROI, we extracted 4096 high-dimensional deep-learning features (Supplementary File.pdf, Supplemental Digital Content, http://links.lww.com/MD/O223). For these deep-learning features, we used the same selection procedures as that for the radiomics features, to obtain the significantly survival-related deep-learning features.

### 2.6. Signature construction and evaluation

To reduce the number of features for model construction without losing any important information, we integrated these features into 2 signatures by LASSO method. One is a fusion signature covering the radiomics and deep-learning features from the rectangular ROI, and another is used for comparison integrating the radiomics features from the exact tumor region. They were then evaluated by Cox proportional hazards regression model (HR [hazard ratio], and *P*-value), Kaplan–Meier analysis (*P*-value), receiver operating characteristic curves (area under curve), and concordance index (C-index).

These 2 signatures were compared to find the most efficient one which might possibly avoid the workloads of radiologists for detailed tumor segmentation. Lastly, we analyzed the performance of the optimal signature in groups classified by clinical factors, to test whether it covered valuable information for survival prognosis beyond the clinical information.

### 2.7. Nomogram development

Considering the effectiveness of the proposed optimal signature beyond the clinical information, we developed a nomogram based on this signature and clinicopathologic risk factors in this part. Its superiority in survival prediction over the clinical nomogram was assessed by C-index and the calibration curves to show the consistency between actual survival ratio and predicted survival probability.

## 3. Results

### 3.1. Clinicopathologic risk factors

In this study, we randomly divided the 670 ccRCC patients in training and test datasets with a ratio of 3:1. The division randomness was validated by ANOVA analysis. It showed no significant differences (*P* > .3) between the training and test cohorts for all the clinical variables, as presented in Table 1. Then the clinicopathologic risk factors were identified by Cox, including “pathologic stage” and “age” with *P*-values of .034 and .006, respectively, in the training dataset, *P*-values of .022 and .031, respectively, in the test dataset and *P*-values of .016 and .043, respectively, in the validation dataset. They were used in the following analysis to show the survival prognosis ability of clinical information.

**Table 1:** Statistical analysis results for clinical factors.

Clinical characteristics	Training (503)		Test (167)		Validation (152)		Training-test‡
	Distribution	*P* a	Distribution	*P* a	Distribution	*P* †	
Age (years) Mean ± SD	59.36 ± 11.30	.006*	58.12 ± 12.32	.031*	59.05 ± 13.27	.043*	0.601
Gender		.772		.856		.993	0.984
Male	330 (65.61%)		103 (61.68%)		94 (61.84%)		
Female	173 (34.39%)		64 (38.32%)		58 (38.16%)		
Pathologic stage		.034*		.022*		.016*	0.670
Stage I	241 (47.91%)		90 (53.89%)		77 (50.66%)		
Stage II	40 (7.95%)		13 (7.78%)		12 (7.79%)		
Stage III	141 (28.03%)		22 (13.17%)		20 (13.16%)		
Stage IV	81 (16.10%)		42 (25.15%)		43 (28.29%)		
Tumor grade		.090		.123		.151	0.477
G1	5 (0.99%)		2 (1.19%)		2 (1.32%)		
G2	162 (32.21%)		60 (35.93%)		61 (40.13%)		
G3	232 (46.12%)		82 (49.10%)		73 (48.02%)		
G4	104 (20.68%)		23 (13.77%)		16 (10.52%)		
Laterality		.317		.295		.340	0.476
Left	262 (52.08%)		88 (52.69%)		79 (51.97%)		
Right	241 (47.91%)		79 (47.31%)		73 (48.03%)		
Survival time (days)	1254.12 ± 684.24	–	1231.59 ± 659.23	–	1251.11 ± 706.71	–	0.601
Survival state		–		–		–	
Alive	350 (69.58%)		110 (65.87%)		101 (66.45%)		0.328
Dead	153 (30.42%)		57 (34.13%)		51 (33.55%)		

* *P* ≤ .05.

† Cox proportional hazard regression model.

‡ ANOVAR test.

### 3.2. Significant features

**Figure 2.**
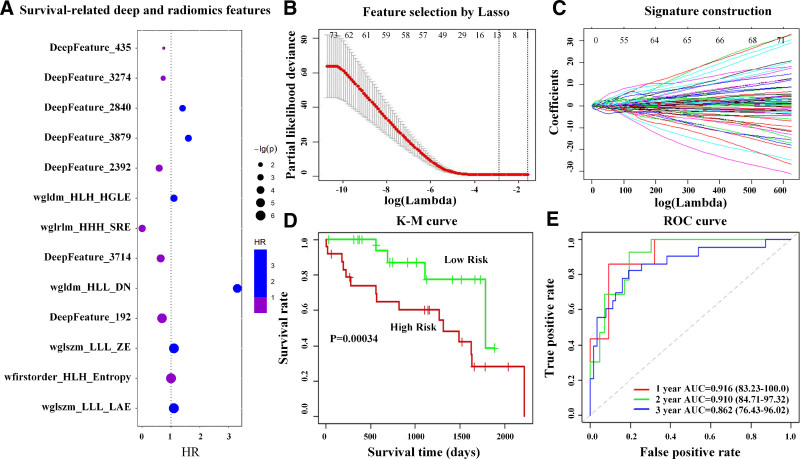
The construction process and evaluation results of the fusion signature from the rectangular region of interest. Significant features were selected by (A) Cox proportional hazard regression model using the criteria of *P* < .05 and (B) the least absolute shrinkage and selection operator (LASSO). (C) Fusion signature construction by LASSO method. (D and E) The assessment of this signature by Kaplan–Meier curves and receiver operating characteristic curves in the test dataset.

After sequential selection process for the features extracted from rectangular ROI (Fig. 2A and B), we identified significantly survival-related features, including 6 wavelet radiomics features and 7 deep-learning features. These significant features described the zone entropy and large area emphasis based on the gray-level size-zone matrix, showed high gray level emphasis and nonuniformity dependence based on the gray level dependence matrix, addressed short run emphasis based on the gray level run length matrix, represented the entropy value of the first-order statistical features, and indicated the high-dimensional abstractions of lesions and their surrounding parenchyma regions. This result revealed the potential roles of wavelet radiomics and deep-learning features in medical image analysis.

Additionally, we selected 5 significantly survival-related radiomics features based on the exact tumor regions (see Fig. S1A and B, Supplemental Digital Content, http://links.lww.com/MD/O224, which illustrates the construction process and evaluation results of the radiomics signature from the exact tumor region). They were all wavelet features, describing the zone entropy and large-area high-gray level emphasis based on the gray-level size-zone matrix, representing the short run emphasis based on the gray level run length matrix, showing the busyness based on the neighboring gray tone difference matrix, or emphasizing the normalized inverse difference calculated on gray level co-occurrence matrix.

### 3.3. Signature construction and evaluation

Based on the 6 wavelet features and 7 deep-learning features from the rectangular ROI, a fusion signature was constructed by the LASSO method (Fig. 2C), as the formula (1) shown in Supplementary File, Supplemental Digital Content, http://links.lww.com/MD/O223. For comparison, a radiomics signature based on the exact tumor region was also defined according to formula (2) in Supplementary File, Supplemental Digital Content, http://links.lww.com/MD/O223.

Both signatures indicate a remarkable prognosis effect on the survival of patients with ccRCC as shown in Table 2. But the fusion signature performed better than the radiomics signature with smaller P, bigger HR, and higher C-index values. The results of Kaplan–Meier curves (Fig. 2D and Fig S1D, Supplemental Digital Content, http://links.lww.com/MD/O224) also support the analysis results above, with more significant differences between the high- and low-risk groups classified by the fusion signature. Besides, ROC curves in Figure 2E and Figure S1E, Supplemental Digital Content, http://links.lww.com/MD/O224 also describe a relatively more accurate prognosis performance for the fusion signature. The results of Kaplan–Meier curves (see Fig. S3A and C, Supplemental Digital Content, http://links.lww.com/MD/O224, which illustrates the performance of the fusion signature based on the CT images in the validation dataset) and ROC curves (see Fig. S3B and D, Supplemental Digital Content, http://links.lww.com/MD/O224, which illustrates the performance of the fusion signature based on the CT images in the validation dataset) in the validation dataset also support the analysis results.

**Table 2:** Comparison of the radiomics signature based on the exact tumor region and the fusion signature based on the rectangular ROI.

Characteristics	Training (503)				Validation (167)				Validation (152)			
	HR	P_COX_	P_KM_	C-index	HR	P_COX_	P_KM_	C-index	HR	P_COX_	P_KM_	C-index
Age	1.036 (1.006–1.067)	0.006	0.017	0.633 ± 0.488	1.027 (0.980–1.075)	0.043	0.027	0.573 ± 0.956	1.022 (0.978–1.086)	0.035	0.028	0.586 ± 0.856
Pathologic stage	1.869 (1.379–2.532)	0.034	5.40e-5	0.697 ± 0.510	2.281 (1.328–3.919)	0.016	0.003	0.759 ± 0.924	2.123 (1.235–3.825)	0.013	0.004	0.748 ± 0.865
Radiomics signature	3.086 (1.400–4.771)	6.00e-4	3.25e-5	0.756 ± 0.08	3.568 (2.929–6.300)	0.008	0.001	0.761 ± 0.094	3.452 (2.825–6.265)	0.007	0.002	0.765 ± 0.065
Fusion signature	4.378 (2.849–6.728)	1.65e-11	8.66e-6	0.912 ± 0.068	5.226 (2.214–12.34)	0.0001	3.4e-4	0.916 ± 0.055	5.352 (2.126–11.23)	0.0002	2.6e-4	0.911 ± 0.052

ROI = region of interest.

**Figure 3.**
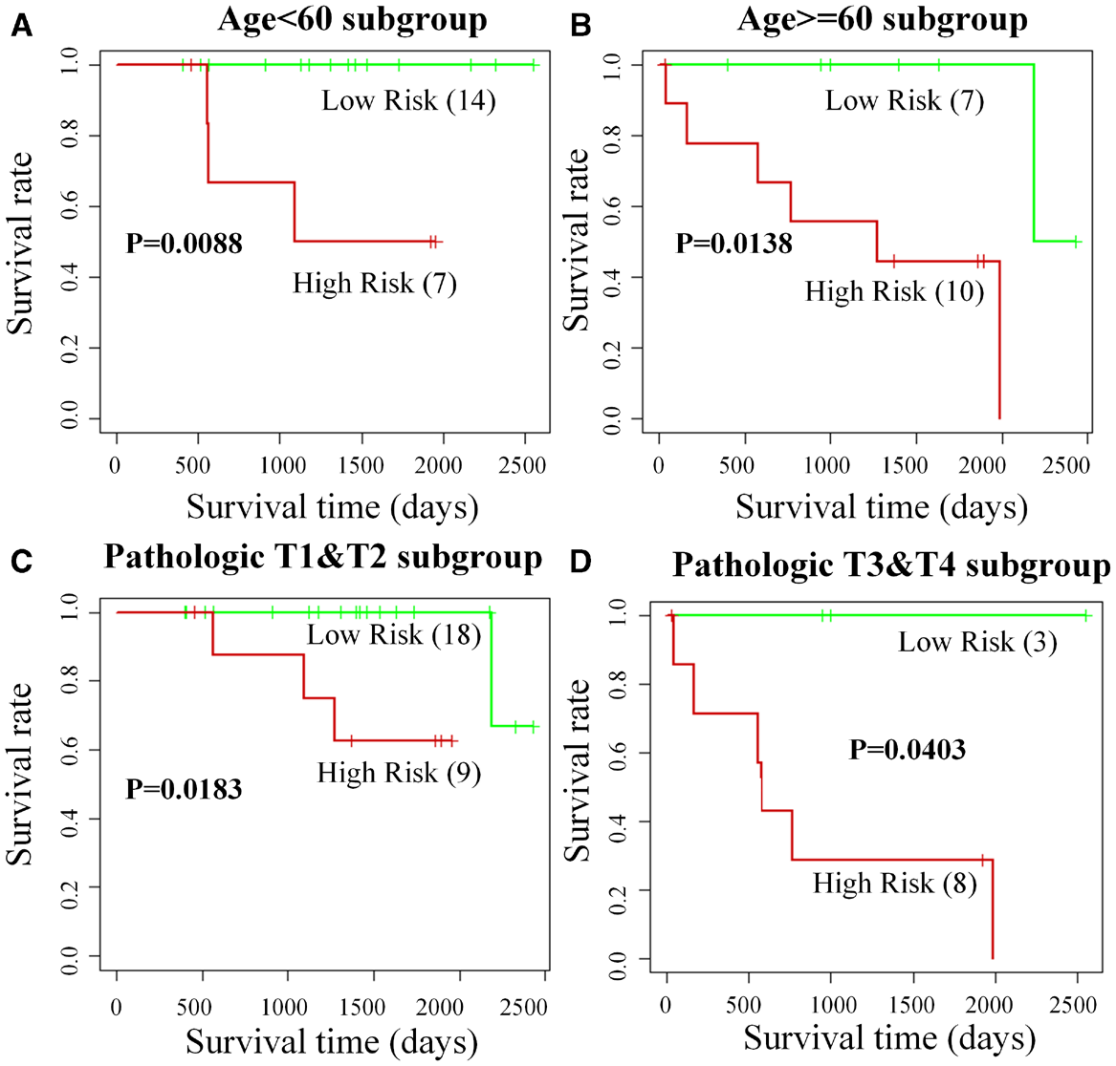
Performance of the fusion signature in different clinical subgroups. (A) Age < 60, (B) age ≥ 60, (C) pathologic T1&T2, and (D) pathologic T3&T4 based on the test dataset. The results for the validation dataset are shown in Figure S2, Supplemental Digital Content, http://links.lww.com/MD/O224.

Additionally, we validated the performance of this fusion signature in different clinical subgroups. This allows us to confirm the necessity of using medical image features beyond clinicopathologic risk factors. The Kaplan–Meier analysis curves are shown in Figure 3 for the test dataset and Figure S2 (see Fig. S2, Supplemental Digital Content, http://links.lww.com/MD/O224, which illustrates the performance of the fusion signature in different clinical subgroups) for the validation dataset. Both figures indicate that the fusion signature could classify the high- and low-risk cohorts precisely even in the same subgroup of “age” or “pathologic stage.”

All the above analyses reveal the superiority of the fusion signature, which can achieve a better prognosis result and avoid the need for radiologists to segment tumor region meticulously.

### 3.4. Nomogram development and assessment

**Figure 4.**
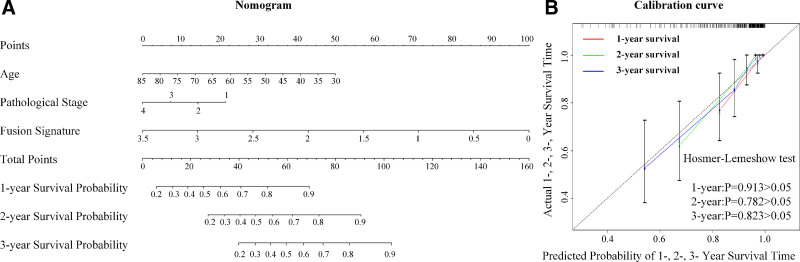
The proposed model for ccRCC survival prognosis. The proposed fusion nomogram was based on the fusion signature and 2 other clinicopathologic risk factors (A). Its performance was assessed by calibration curves (B).

Given the efficient contributions of the fusion signature to the survival prognosis of ccRCC, we integrated it with 2 other clinicopathologic risk factors, “age” and “pathologic tumor stage,” to generate a fusion nomogram based on the training cohort, as displayed in Figure 4A.

The C-index results of this nomogram are 0.916 (95% [CI]: 0.861–0.971) in the test cohort, and 0.911 (95% [CI]: 0.859–0.963) in the validation cohort. The performance is much better than the clinical nomogram containing only 2 clinicopathologic risk factors, which has a C-index of 0.738 (95% [CI]: 0.646–0.830) in the test dataset and 0.743 (95% [CI]: 0.616–0.870) in the validation dataset. Likewise, the calibration curves of the fusion nomogram in Figure 4B show that the predicted survival probability is very close to its actual rate (Hosmer–Lemeshow test, 1-year: *P* = .913, 2-year: *P* = .782, 3-year: *P* = .823). Therefore, this fusion nomogram may provide an alternative way to predict the survival of patients with ccRCC in future clinical use.

## 4. Discussion

On the survival prognosis of ccRCC, several previous literature studied the effects of clinical and treatment factors,^[20]^ immune cell,^[21,22]^ tumor enhancement in the nephrographic phase of CT images,^[23]^ tumor size and the presence of renal vein invasion from CT images,^[24]^ shape features from histopathology images,^[25,26]^ and gene expressions.^[27,28]^

Of these studies, 2^[27,28]^ paying attention to gene expressions in the survival prognosis of ccRCC patients did not achieve a better performance than ours (ROC: 0.862 vs 0.776, 0.862 vs 0.755). In addition, 3 other studies^[23,24]^ validated the importance of the features extracted from CT images in the field of survival prediction for ccRCC patients. Since CT scans are routine procedures for ccRCC patients in hospitals, clinicians do not need to do additional procedures to use the CT image-based tools. The first 2 studies investigated a few CT image features including tumor size, presence of vein invasion, and tumor enhancement which significantly were associated with survival. Specially, the last study is similar to ours. However, our study proposed a signature based on the best contrast-enhanced CT image, not all enhanced and non-enhanced CT images. Moreover, our signature was calculated on 2 dimensional rectangular ROI rather than 3D exact tumor regions. It means that our signature could reduce the radiologists’ workloads in tumor segmentation and also avoid the needs of more CT scans. Thus, this signature is useful to predict the survival probability for patients with ccRCC in future clinical cases.

The proposed fusion signature included 6 traditional wavelet features and 7 deep-learning features. These characteristics are all significantly related to the survival of ccRCC patients tested by the Cox. It indicates that the wavelet-based and deep-learning features contain valuable information for survival prediction, which is consistent with previous studies.^[29–31]^

Compared to the radiomics signature based on the exact tumor region, this fusion signature obtained better evaluation results in ccRCC survival prognosis across the indexes including HR, P_COX_, P_KM_, area under curve, and C-index. More importantly, it could be easily achieved since it does not require radiologists to perform detailed tumor segmentation. Additionally, it was also validated to contain valuable information beyond the clinicopathologic risk factors. All these analyses indicate that this signature is applicable and valuable in the survival prognosis of ccRCC patients.

Thus, the fusion nomogram integrating this signature and 2 other clinicopathologic risk factors showed a better performance with an increase of about 20% in the C-index results than the clinical nomogram in both test and validation datasets. Also, the consistency of the survival probability predicted by the proposed nomogram and the actual ratio indicates its usefulness in future clinical use.

## 5. Conclusions

The proposed fusion signature shows more accurate prognosis performance for ccRCC patients without the requirement to perform a detailed segmentation for radiologists. This signature, combined with clinicopathologic risk factors provided a noninvasive and easy-to-use model to predict the survival probability for patients with ccRCC in future clinical use.

## Acknowledgments

We thank the members of the School of Life Science and Technology for valuable discussions.

## Author contributions

**Conceptualization:** Yu Yan.

**Data curation:** Tao Wang, Yu Yan.

**Formal analysis:** Meng Zhou.

**Supervision:** Xiaobo Zhou, Liyu Huang.

**Writing – original draft:** Zhennan Lu, Sijia Wu.

**Writing – review & editing:** Dan Ni.

## Supplementary Material



## References

[1] Bezhanova SD. Tumors of the kidney. The new 2016 WHO classification of tumors of the genitourinary system. Arkh Patol. 2017;79:48–52. Opukholi pochek. Novaia klassifikatsiia opukholeĭ urogenital’noĭ sistemy Vsemirnoĭ organizatsii zdravookhraneniia 2016 g. 10.17116/patol201779248-52 28418358

[2] Feng X Zhang L Tu W Cang S. Frequency, incidence and survival outcomes of clear cell renal cell carcinoma in the United States from 1973 to 2014: a SEER-based analysis. Medicine (Baltimore). 2019;98:e16684. 31374051 10.1097/MD.0000000000016684 PMC6708618

[3] Cheville JC Lohse CM Zincke H Weaver AL Blute ML. Comparisons of outcome and prognostic features among histologic subtypes of renal cell carcinoma. Am J Surg Pathol. 2003;27:612–24. 12717246 10.1097/00000478-200305000-00005

[4] Gudbjartsson T Hardarson S Petursdottir V Thoroddsen A Magnusson J Einarsson GV. Histological subtyping and nuclear grading of renal cell carcinoma and their implications for survival: a retrospective nation-wide study of 629 patients. Eur Urol. 2005;48:593–600. 15964127 10.1016/j.eururo.2005.04.016

[5] Leibovich BC Lohse CM Cheville JC. Predicting oncologic outcomes in renal cell carcinoma after surgery. Eur Urol. 2018;73:772–80. 29398265 10.1016/j.eururo.2018.01.005

[6] Zheng Z Chen Z Xie Y Zhong Q Xie W. Development and validation of a CT-based nomogram for preoperative prediction of clear cell renal cell carcinoma grades. Eur Radiol. 2021;31:6078–86. 33515086 10.1007/s00330-020-07667-y

[7] Wang R Hu Z Shen X. Computed tomography-based radiomics model for predicting the WHO/ISUP grade of clear cell renal cell carcinoma preoperatively: a multicenter study. Front Oncol. 2021;11:543854. 33718124 10.3389/fonc.2021.543854 PMC7946982

[8] Kocak B Durmaz ES Ates E Ulusan MB. Radiogenomics in clear cell renal cell carcinoma: machine learning-based high-dimensional quantitative CT texture analysis in predicting PBRM1 mutation status. AJR Am J Roentgenol. 2019;212:W55–63. 30601030 10.2214/AJR.18.20443

[9] He X Wei Y Zhang H. Grading of clear cell renal cell carcinomas by using machine learning based on artificial neural networks and radiomic signatures extracted from multidetector computed tomography images. Acad Radiol. 2020;27:157–68. 31147235 10.1016/j.acra.2019.05.004

[10] Shu J Tang Y Cui J. Clear cell renal cell carcinoma: CT-based radiomics features for the prediction of Fuhrman grade. Eur J Radiol. 2018;109:8–12. 30527316 10.1016/j.ejrad.2018.10.005

[11] Uhm KH Jung SW Choi MH. Deep learning for end-to-end kidney cancer diagnosis on multi-phase abdominal computed tomography. NPJ Precis Oncol. 2021;5:54. 34145374 10.1038/s41698-021-00195-y PMC8213852

[12] Lin F Ma C Xu J. A CT-based deep learning model for predicting the nuclear grade of clear cell renal cell carcinoma. Eur J Radiol. 2020;129:109079. 32526669 10.1016/j.ejrad.2020.109079

[13] Coy H Hsieh K Wu W. Deep learning and radiomics: the utility of Google TensorFlow™ Inception in classifying clear cell renal cell carcinoma and oncocytoma on multiphasic CT. Abdom Radiol (NY). 2019;44:2009–20. 30778739 10.1007/s00261-019-01929-0

[14] Han S Hwang SI Lee HJ. The classification of renal cancer in 3-phase CT images using a deep learning method. J Digit Imaging. 2019;32:638–43. 31098732 10.1007/s10278-019-00230-2 PMC6646616

[15] Sheth S Fishman EK. Multi-detector row CT of the kidneys and urinary tract: techniques and applications in the diagnosis of benign diseases. Radiographics. 2004;24:e20. 14730056 10.1148/rg.e20

[16] Fedorov A Beichel R Kalpathy-Cramer J. 3D Slicer as an image computing platform for the quantitative imaging network. Magn Reson Imaging. 2012;30:1323–41. 22770690 10.1016/j.mri.2012.05.001 PMC3466397

[17] Davnall F Yip CS Ljungqvist G. Assessment of tumor heterogeneity: an emerging imaging tool for clinical practice? Insights Imaging. 2012;3:573–89. 23093486 10.1007/s13244-012-0196-6 PMC3505569

[18] Thibault G Angulo J Meyer F. Advanced statistical matrices for texture characterization: application to cell classification. IEEE Trans Biomed Eng. 2014;61:630–7. 24108747 10.1109/TBME.2013.2284600

[19] Dawud AM Yurtkan K Oztoprak H. Application of deep learning in neuroradiology: brain haemorrhage classification using transfer learning. Comput Intell Neurosci. 2019;2019:4629859. 31281335 10.1155/2019/4629859 PMC6589279

[20] Dabestani S Beisland C Stewart GD. Long-term outcomes of follow-up for initially localised clear cell renal cell carcinoma: RECUR database analysis. Eur Urol Focus. 2019;5:857–66. 29525381 10.1016/j.euf.2018.02.010

[21] Selvi I Demirci U Bozdogan N Basar H. The prognostic effect of immunoscore in patients with clear cell renal cell carcinoma: preliminary results. Int Urol Nephrol. 2020;52:21–34. 31541404 10.1007/s11255-019-02285-0

[22] Su S Akbarinejad S Shahriyari L. Immune classification of clear cell renal cell carcinoma. Sci Rep. 2021;11:4338. 33619294 10.1038/s41598-021-83767-z PMC7900197

[23] Maehara J Nishie A Asayama Y. Tumor enhancement on dynamic CT: a predictive factor for recurrence after nephrectomy in localized T1 clear cell renal cell carcinoma. Anticancer Res. 2018;38:2377–83. 29599364 10.21873/anticanres.12486

[24] Hötker AM Karlo CA Zheng J. Clear cell renal cell carcinoma: associations between CT features and patient survival. AJR Am J Roentgenol. 2016;206:1023–30. 26934514 10.2214/AJR.15.15369 PMC4983700

[25] Tabibu S Vinod PK Jawahar CV. Pan-renal cell carcinoma classification and survival prediction from histopathology images using deep learning. Sci Rep. 2019;9:10509. 31324828 10.1038/s41598-019-46718-3 PMC6642160

[26] Chen S Zhang N Jiang L. Clinical use of a machine learning histopathological image signature in diagnosis and survival prediction of clear cell renal cell carcinoma. Int J Cancer. 2021;148:780–90. 32895914 10.1002/ijc.33288

[27] Berglund A Amankwah EK Kim YC. Influence of gene expression on survival of clear cell renal cell carcinoma. Cancer Med. 2020;9:8662–75. 32986937 10.1002/cam4.3475 PMC7666730

[28] Liu Y Huang Z Cheng G. Development of a four-gene prognostic model for clear cell renal cell carcinoma based on transcriptome analysis. Genomics. 2021;113:1816–27. 33838279 10.1016/j.ygeno.2021.04.005

[29] Lao J Chen Y Li ZC. A deep learning-based radiomics model for prediction of survival in glioblastoma multiforme. Sci Rep. 2017;7:10353. 28871110 10.1038/s41598-017-10649-8 PMC5583361

[30] Yang L Yang J Zhou X. Development of a radiomics nomogram based on the 2D and 3D CT features to predict the survival of non-small cell lung cancer patients. Eur Radiol. 2019;29:2196–206. 30523451 10.1007/s00330-018-5770-y

[31] Zhang Y Lobo-Mueller EM Karanicolas P Gallinger S Haider MA Khalvati F. Improving prognostic performance in resectable pancreatic ductal adenocarcinoma using radiomics and deep learning features fusion in CT images. Sci Rep. 2021;11:1378. 33446870 10.1038/s41598-021-80998-y PMC7809062

