# Supplementary material for: Survival analysis of clear cell renal cell carcinoma based on radiomics and deep learning features from CT images
Source: Medicine (Baltimore). 2024 Dec 20;103(51):e40723. doi: 10.1097/MD.0000000000040723 (PMC11666162; doi:10.1097/MD.0000000000040723)
Supplement: Supplementary file 1 [file medi-103-e40723-s001.pdf]

Survival Analysis of Clear Cell Renal Cell Carcinoma Based on Radiomics and Deep Learning Features from CT Images

Supplementary Material

1 Supplemental Formulas

1. Fusion signature based on the rectangular ROI for CT images

$$\begin{aligned} &FusionSignature \\ &= -0.6383 \times R_{wglrlm\_HHH\_SHE} - 0.0625 \times R_{wfirstorder\_HLH\_Entropy} + 0.5359 \times R_{wgldm\_HLL\_DN} \\ &\quad + 2.2745 \times R_{wglszm\_LLL\_LAE} + 0.1756 \times R_{wglszm\_LLL\_ZoneEntropy} + 0.8473 \times R_{wgldm\_HLH\_HGLE} \\ &\quad - 0.1014 \times Deep_{192} - 0.0049 \times Deep_{435} - 0.0352 \times Deep_{2392} - 0.2094 \times Deep_{2840} \\ &\quad - 0.0996 \times Deep_{3274} - 1.172 \times Deep_{3714} - 0.3755 \times Deep_{3879} \end{aligned} \quad (1)$$

2. Radiomics signature based on the exact tumor region

$$\begin{aligned} &RadomicsSignature \\ &= 1.961 \times R_{wglcm\_HHL\_IDN} + 1.2263 \times R_{wglrlm\_LLL\_LRE} + 1.2868 \times R_{wngtdm\_LHL\_Busyness} \\ &\quad + 0.0523 \times R_{wglszm\_LLL\_ZoneEntropy} + 0.913 \times R_{wglszm\_HLL\_LAHGLE} \end{aligned} \quad (2)$$

2 The process to extract the radiomics features

For the rectangular ROI and the exact tumor region, we used "pyradiomics" (https://pyradiomics. readthedocs.io/, v3.6.0) to extract the radiomics features. This tool has been used in multiple previous studies[1][2][3][4]. The features covered (1) first-order statistical features describing the gray-scale distribution of pixels, (2) shape features quantifying the shape and size of the tumor area, (3) textural features estimating the uniformity, heterogeneity, coarse degree, etc., and (4) wavelet features calculated on the images preprocessed using wavelet filters.

Procedures to extract radiomics features from CT images by pyradiomics:

(1) ROI was determined from original CT images and the masked images drew by radiologists.

(2) ROI images were transformed to wavelet images by pywavelets.

(3) Radiomics features including wavelet features were extracted from the ROI images by pyradiomics.

We used the following formulas to calculate the features involved in the signatures (1-2).

First order features

$$Entropy = -\sum_{i=1}^{N_g} p(i) log_2 (p(i) + \varepsilon) \tag{3}$$

GLSZM features (LAE: Large Area Emphasis, SAE: Small Area Emphasis, LAHGLE: Large Area High Gray Level Emphasis)

$$LAE = \frac{\sum_{i=1}^{N_g}\sum_{j=1}^{N_s} P(i,j) j^2}{N_z} \tag{4}$$

$$SAE = \frac{\sum_{i=1}^{N_g}\sum_{j=1}^{N_s} \frac{P(i,j)}{j^2}}{N_z} \tag{5}$$

$$LANHGLE = \frac{\sum_{i=1}^{N_g}\sum_{j=1}^{N_s} P(i,j) i^2 j^2}{N_z} \tag{6}$$

$$Zone\ Entropy = -\sum_{i=1}^{N_g}\sum_{j=1}^{N_s} p(i,j) log_2\left(p(i,j)+\varepsilon\right) \tag{7}$$

GLDM features (DN: Dependence Non-Uniformity, HGLE: High Gray Level Emphasis)

$$DN = \frac{\sum_{j=1}^{N_d}\left(\sum_{i=1}^{N_g} P(i,j)\right)^2}{N_z} \tag{8}$$

$$HGLE = \frac{\sum_{i=1}^{N_g}\sum_{j=1}^{N_d} P(i,j)i^2}{N_z} \tag{9}$$

GLRLM features (LRE: Long Run Emphasis, SRE:Short Run Emphasis)

$$SRE = \frac{\sum_{i=1}^{N_g}\sum_{j=1}^{N_r} \frac{P(i,j|\ \theta)i^2}{j^2}}{N_r(\theta)} \tag{10}$$

$$LRE = \frac{\sum_{i=1}^{N_g}\sum_{j=1}^{N_r} \frac{P(i,j|\ \theta)j^2}{j^2}}{N_r(\theta)} \tag{11}$$

GLCM features (IDN: Inverse Difference Normalized)

$$IDN = \sum_{k=0}^{N_g-1} \frac{p_{x-y}(k)}{1+(\frac{k}{N_g})} \tag{12}$$

3 The process to extract the deep-learning features

The pre-trained Alexnet framework[5] for the extraction of deep-learning features has validated its effectiveness in a series of medical image problems, such as detection of abnormal brain[6], overall survival prediction for brain tumor patients[7], and histopathology image classification, segmentation segmentation , and visualization[8]. Thus, for the resized square ROI images with a size of 227×227, we removed the last fully connected layer and used the other layers from the pre-trained Alextnet framework to extract the deep-learning features involved in our study. The pre-trained Alexnet framework was downloaded from GitHub (https://github.com/kratzert/finetune_alexnet_with_tensorflow/tree/5d751d62eb4d7149f4e3fd465febf8f07d4cea9d).

4 The process to select features

(1) We used cox proportional hazard regression model to select features significantly related to ccRCC survival ($P<0.05$).

(2) The least absolute shrinkage and selection operator (LASSO) method was used further to select more survival related features. 10-fold cross validation with 100 resampling was conducted in this process to improve the expansion ability of these features.

(3) All survival related features were ranked according to the occurrence during the resampling. The top features were used to establish the signature by LASSO method.

5 Supplemental Figures

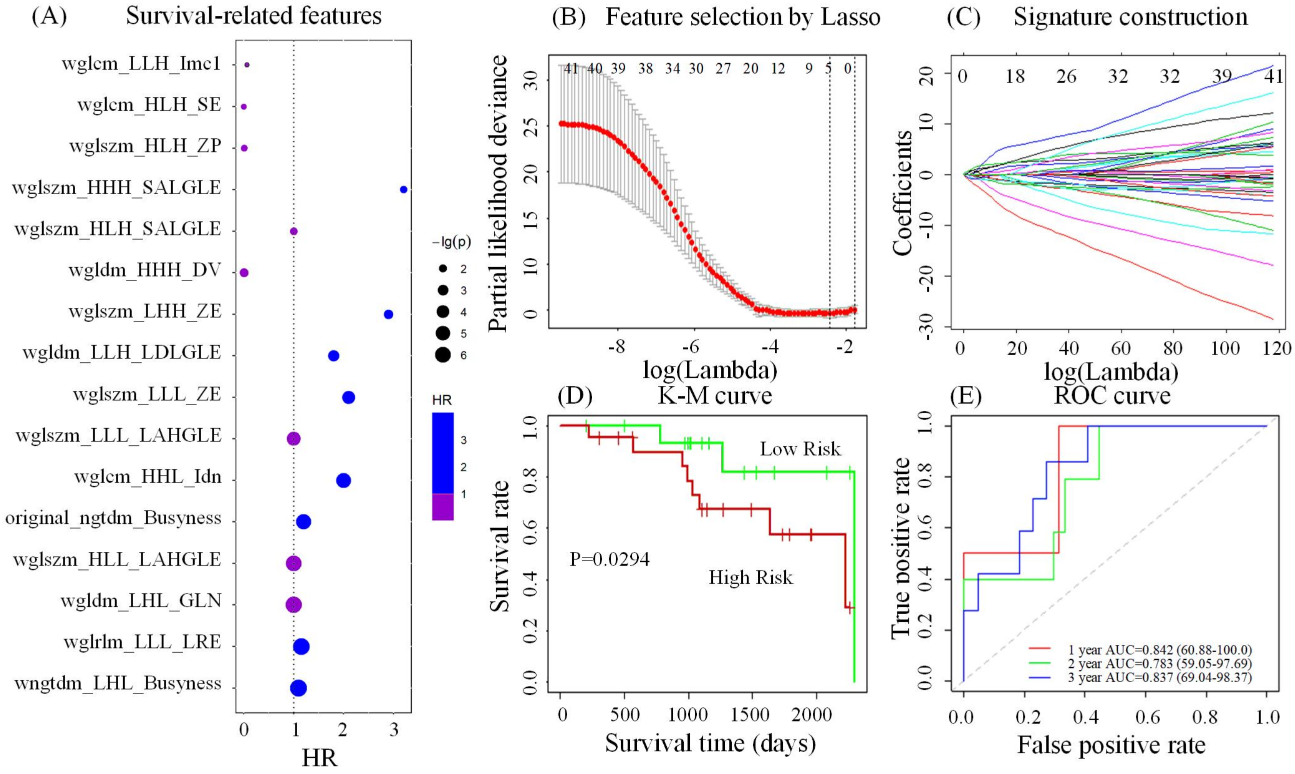

Supplemental Figure 1. The construction process and evaluation results of the radiomics signature from the exact tumor region. Significant features were selected by (A) Cox proportional hazard regression model using the criteria of $p<0.05$ and (B) the least absolute shrinkage and selection operator (LASSO). (C) Radiomics signature construction by LASSO method. (D-E) The assessment of this signature by Kaplan-Meier curves and receiver operating characteristic curves in the validation dataset.

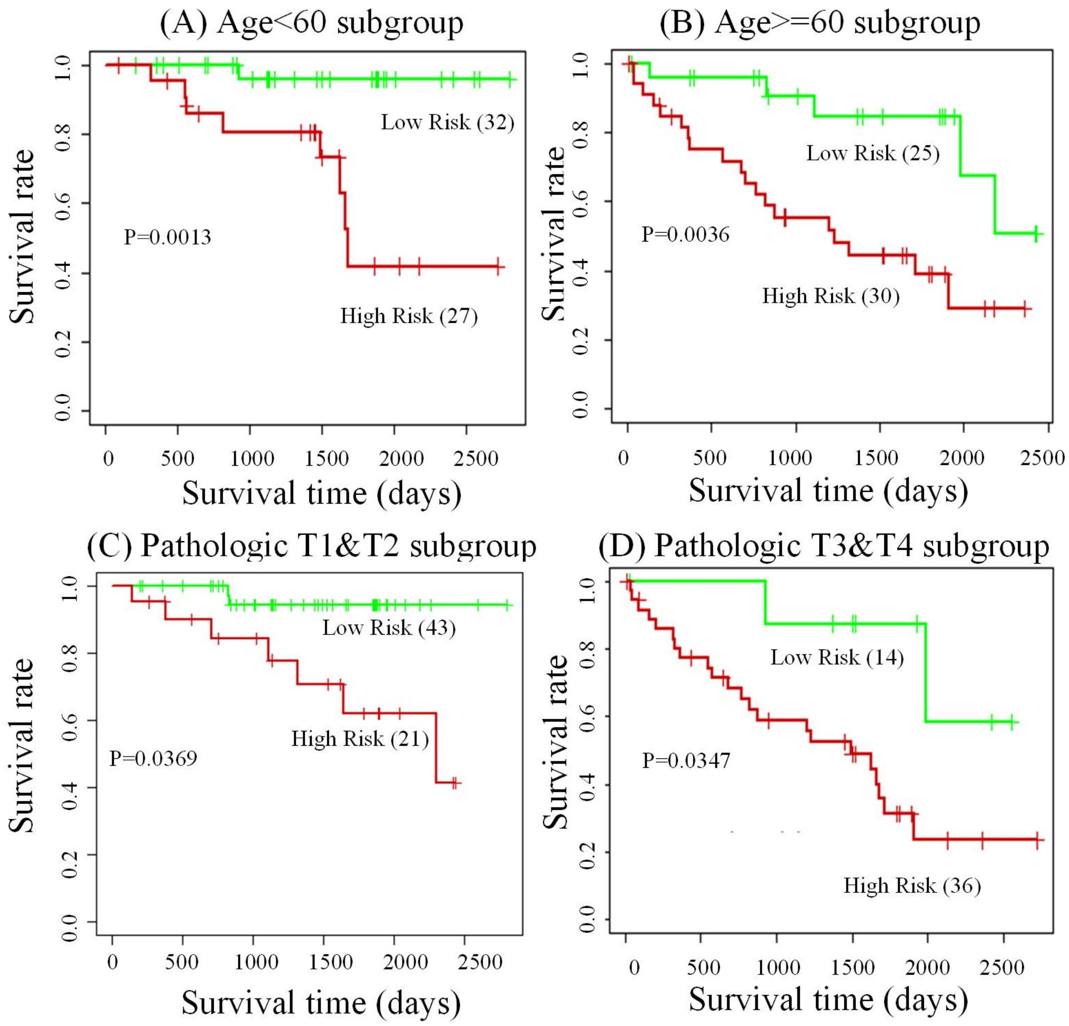

Supplemental Figure 2. Performance of the fusion signature in different clinical subgroups: (A) Age<60, (B) Age>=60, (C) Pathologic T1&T2 and (D) Pathologic T3&T4 based on the validation dataset.

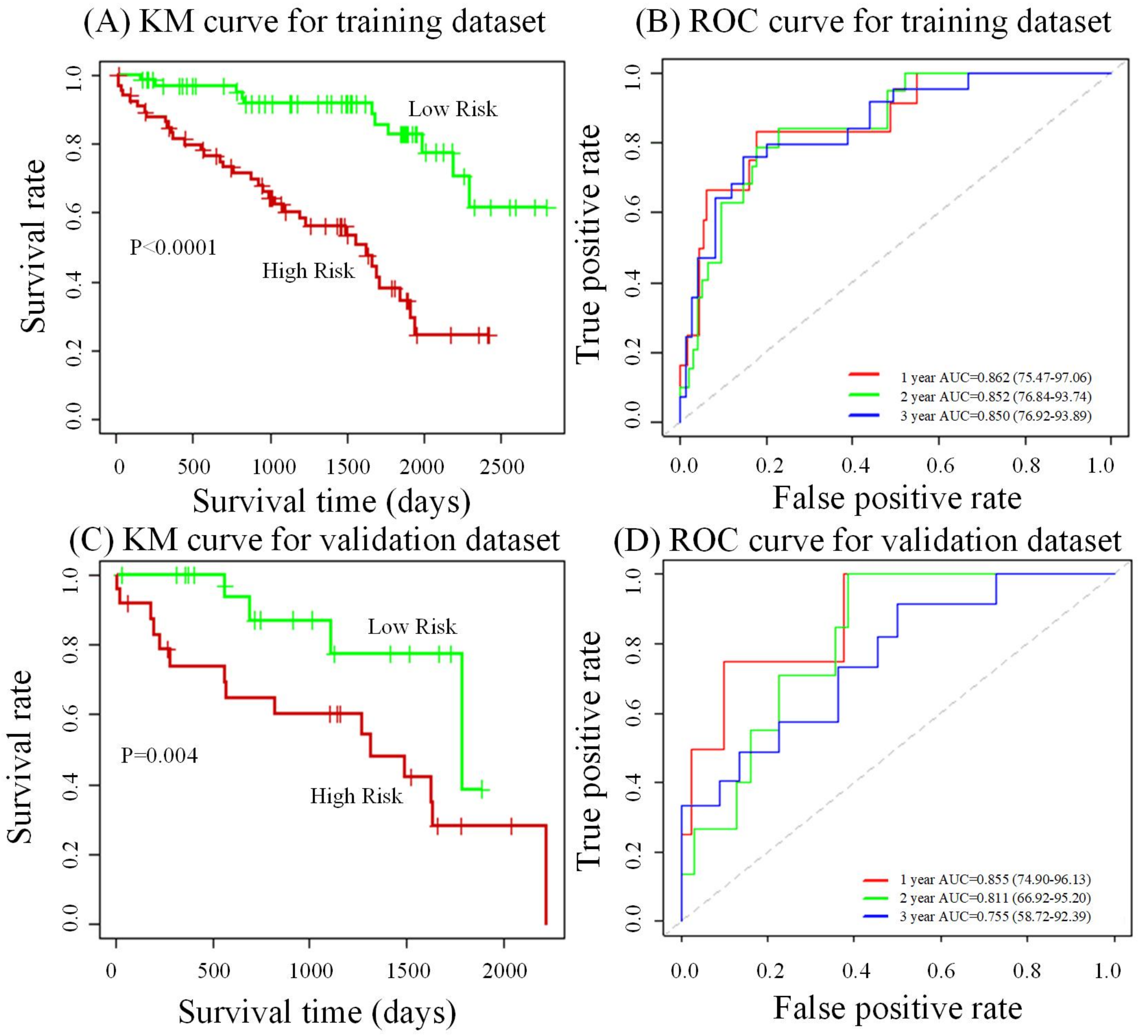

Supplemental Figure 3. Performance of the fusion signature based on the CT images in the validation dataset. This signature was assessed by Kaplan-Meier (K-M) curves (A) and receiver operating characteristic (ROC) curves (B) from the rectangular region of interest, and by K-M curves (C) and ROC curves (D) from the exact tumor region.

6 References

[1] Nazari, M., Shiri, I., & Zaidi, H. (2021). Radiomics-based machine learning model to predict risk of death within 5-years in clear cell renal cell carcinoma patients. Computers in biology and medicine, 129, 104135. https://doi.org/10.1016/j.compbiomed.2020.104135.

[2] van Griethuysen, J., Fedorov, A., Parmar, C., Hosny, A., Aucoin, N., Narayan, V., Beets-Tan, R., Fillion-Robin, J. C., Pieper, S., & Aerts, H. (2017). Computational Radiomics System to Decode the Radiographic Phenotype. Cancer research, 77(21), e104–e107. https://doi.org/10.1158/0008-5472.CAN-17-0339.

[3] Ran, J., Cao, R., Cai, J., Yu, T., Zhao, D., & Wang, Z. (2021). Development and Validation of a Nomogram for Preoperative Prediction of Lymph Node Metastasis in Lung Adenocarcinoma Based on Radiomics Signature and Deep Learning Signature. Frontiers in oncology, 11, 585942. https://doi.org/10.3389/fonc.2021.585942.

[4] De Araujo Faria, V., Azimbagirad, M., Viani Arruda, G., Fernandes Pavoni, J., Cezar Felipe, J., Dos Santos, E., & Murta Junior, L. O. (2021). Prediction of Radiation-Related Dental Caries Through PyRadiomics Features and Artificial Neural Network on Panoramic Radiography. Journal of digital imaging, 34(5), 1237–1248. https://doi.org/10.1007/s10278-021-00487-6.

[5] Krizhevsky, A., Sutskever, I., and Hinton, G.E. (2017). Imagenet classification with deep convolutional neural networks. Communications of the ACM 60, 84-90.

[6] Lu, S., Wang, SH. and Zhang, YD.(2021). Detection of abnormal brain in MRI via improved AlexNet and ELM optimized by chaotic bat algorithm. Neural Comput & Applic 33, 10799– 10811.

[7] Chato, L., and Latifi, S. (Year). "Machine learning and deep learning techniques to predict overall survival of brain tumor patients using MRI images", in: 2017 IEEE 17th International Conference on Bioinformatics and Bioengineering (BIBE): IEEE), 9-14.

[8] Xu, Y., Jia, Z., Wang, L. B., Ai, Y., Zhang, F., Lai, M., & Chang, E. I. (2017). Large scale tissue histopathology image classification, segmentation, and visualization via deep convolutional activation features. BMC bioinformatics, 18(1), 281. https://doi.org/10.1186/s12859-017-1685-x.
