# Supplementary material for: Survival analysis of clear cell renal cell carcinoma based on radiomics and deep learning features from CT images
Source: Medicine (Baltimore). 2024 Dec 20;103(51):e40723. doi: 10.1097/MD.0000000000040723 (PMC11666162; doi:10.1097/MD.0000000000040723)
Supplement: Supplementary file 2 [file medi-103-e40723-s002.docx]

Supplementary Material

# Supplementary Figure 1





**Supplementary Figure 1.** The construction process and evaluation results of the radiomics signature from the exact tumor region. Significant features were selected by (A) Cox proportional hazard regression model using the criteria of p<0.05 and (B) the least absolute shrinkage and selection operator (LASSO). (C) Radiomics signature construction by LASSO method. (D-E) The assessment of this signature by Kaplan-Meier curves and receiver operating characteristic curves in the validation dataset.

Supplementary Material

# Supplementary Figure 2





**Supplementary Figure 2.** Performance of the fusion signature in different clinical subgroups: (A) Age<60, (B) Age>=60, (C) Pathologic T1&T2 and (D) Pathologic T3&T4 based on the validation dataset.

Supplementary Material

# Supplementary Figure 3

**

**

**Supplementary Figure 3.** Performance of the fusion signature based on the CT images in the validation dataset. This signature was assessed by Kaplan-Meier (K-M) curves (A) and receiver operating characteristic (ROC) curves (B) from the rectangular region of interest, and by K-M curves (C) and ROC curves (D) from the exact tumor region.
